# Trapped by habitat choice: Ecological trap emerging from adaptation in an evolutionary experiment

**DOI:** 10.1111/eva.12937

**Published:** 2020-03-28

**Authors:** Frederik Mortier, Dries Bonte

**Affiliations:** ^1^ Terrestrial Ecology Unit Department of Biology Ghent University Ghent Belgium

**Keywords:** adaptation, eco‐evolutionary dynamics, ecological trap, habitat choice, habitat selection, maladaptation, plant–herbivory interactions

## Abstract

Individuals moving in heterogeneous environments can improve their fitness considerably by habitat choice. Induction by past exposure, genetic preference alleles and comparison of local performances can all drive this decision‐making process. Despite the importance of habitat choice mechanisms for eco‐evolutionary dynamics in metapopulations, we lack insights on the connection of their cue with its effect on fitness optimization. We selected a laboratory population of *Tetranychus urticae* Koch (two‐spotted spider mite) according to three distinct host‐choice selection treatments for ten generations. Additionally, we tested the presence of induced habitat choice mechanisms and quantified the adaptive value of a choice before and after ten generations of artificial selection in order to gather insight on the habitat choice mechanisms at play. Unexpectedly, we observed no evolution of habitat choice in our experimental system: the initial choice of cucumber over tomato remained. However, this choice became maladaptive as tomato ensured a higher fitness at the end of the experiment. Furthermore, a noteworthy proportion of induced habitat choice can modify this ecological trap depending on past environments. Despite abundant theory and applied relevance, we provide the first experimental evidence of an emerging trap. The maladaptive choice also illustrates the constraints habitat choice has in rescuing populations endangered by environmental challenges or in pest control.

## 1 INTRODUCTION

Dispersal is an impactful ecological process that connects local populations and their dynamics in space (Hanski, 2012). Dispersal is a decision‐making process at the departure, transfer and settlement stage (Clobert, Le Galliard, Cote, Meylan, & Massot, 2009), that is in part genetically determined (Bonte & Dahirel, 2017; Saastamoinen et al., 2017). Therefore, all three stages of the dispersal process are subjected to evolution. While most dispersal research to date focused on the departure phase, insights in the ecological and evolutionary processes that drive settlement are scarce. Habitat choice, the nonrandomness in who is dispersing (departure), and where to disperse to (settlement; Clobert et al., 2009), introduces directionality in the distribution of certain genotypes and enables them to increase individual fitness (Edelaar & Bolnick, 2019). It can interfere with the homogenizing effect of dispersal on spatial genetics (Berner & Thibert‐Plante, 2015; Bolnick & Otto, 2013; Ravigné, Olivieri, & Dieckmann, 2004; Rice & Salt, 1990; Richardson, Urban, Bolnick, & Skelly, 2014). Furthermore, habitat choice can have far reaching eco‐evolutionary consequences, from increasing adaptation enabling the evolution of ecological specialization in the face of intense gene flow (Armsworth, 2009; Bolnick et al., 2009; Jacob et al., 2017; Ravigné, Dieckmann, & Olivieri, 2009), stabilizing metapopulation dynamics (Mortier, Jacob, Vandegehuchte, & Bonte, 2018) to driving speciation (Berlocher & Feder, 2002; Maynard Smith, 1966; Nicolaus & Edelaar, 2018). Habitat choice likely evolves as an adaptation and should rescue a population from elimination in a changing environment. In essence, habitat choice can be both cause and effect in eco‐evolutionary change rendering it a small, but important cog in the big eco‐evolutionary machinery.

Fast environmental change can disconnect a habitat preference cue from its former fitness advantage, resulting in a maladaptive choice. This transforms habitat choice from an ecological opportunity to an ecological trap (Hale & Swearer, 2016; Robertson & Hutto, 2006; Singer & Parmesan, 2018). Most likely, preference for a certain cue evolved in an environment where following that cue was adaptive. However, a change in the environment can alter the connection between cue and habitat and/or change fitness prospects across different habitat. This could make an organism prefer suboptimal habitats (Robertson & Hutto, 2006; Sih, 2013). For example, Kriska, Malik, and Szivák (2008) report caddis flies choosing unsuitable glass panes to copulate and oviposit on. Polarized light reflected by the glass is a cue that is associated with water in a natural habitat, a very suitable option to oviposit. In fact, many populations in nature occupy suboptimal habitat (Hereford, 2009), which can be explained by an ecological trap as well as constraints such as unawareness of more optimal habitat or the inability to reach more optimal habitat. Unfortunately, researchers have been quick to attribute this to an ecological trap without critical information on the individual choices involved and on the choice behaviour before the onset of the ecological trap (Hale & Swearer, 2016; Robertson & Hutto, 2006; but see Singer & Parmesan, 2018). Understanding the drivers behind maladaptive processes, like ecological traps, is as important as understanding adaptive processes to better understand evolution's impact on conservation, agriculture and other ecology‐driven systems (Brady et al., 2019; Derry et al., 2019).

An individual's behaviour in terms of nonrandom settlement is the combination of all its different preferences applied to whatever habitat the individual perceives to be available. These processes eventually result in a specific habitat choice. Henceforth, we will talk about habitat preference as the individual trait that leads to a tendency to follow a certain cue and which has evolved. The habitat preferences are the mechanisms behind habitat choice, historically referred to as habitat choice mechanisms, that indicate the tendencies to prefer one option above the other when given the choice. We explicitly refer to habitat choice as the decision resulting from all combined habitat preferences among the available habitats in the environment. Habitat preference can follow different types of particular environmental and physiological cues (Akcali & Porter, 2017), which can work simultaneously, in synergy or antagonistically, to dictate a net choice (Camacho, Canal, & Potti, 2016). In brief, we can divide these habitat choice mechanisms in three categories, as discussed in Akcali and Porter (2017): (a) induced habitat choice mechanisms, (b) direct genetic habitat choice mechanisms and (c) matching habitat choice mechanisms. First, an induced habitat choice leads a disperser to be more likely to choose habitat it experienced in the past. Oftentimes, this means a preference for the environment in which the disperser developed (see “habitat imprinting” and “natal habitat preference induction”), reversible or not (see “habitat imprinting”). Induced habitat choice is adaptive when the chosen habitat is suitable, indicating that the individual is adapted to that habitat. This is reasonable considering the individual already experienced it before without noteworthy harm. Although plastic in nature, the strength and direction of plasticity in this environmentally induced choice can be subject to natural selection, for example the Baldwin effect (Crispo, 2007; Simpson, 1953), and can evolve relatively easy (Berner & Thibert‐Plante, 2015). This means that phenotypes can evolve to prefer previously experienced habitat to a greater or lesser extent. Second, a direct genetic habitat choice results in a direct choice for a particular habitat through preferring an ingrained cue. While all habitat choice mechanisms should have a genetic basis to be able to evolve, direct genetic habitat choice forms a direct link between distinct habitat preference alleles and the preferred habitat which is unaffected by other factors like past experiences or fitness prospects. A direct genetic habitat choice is usually evolutionary conservative and only expected to evolve in a narrow range of circumstances in cases of strong selection against not ending up in that specific habitat (Berner & Thibert‐Plante, 2015), for instance in highly specialized parasites. Third, with a matching habitat choice individuals choose habitat which they assess to be most adapted to (Edelaar, Siepielski, & Clobert, 2008). This implies that, as opposed to the learned and inherent habitat cues of both categories above, a direct fitness assessment is the habitat cue in matching habitat choice. To be able to make such a choice at settlement, individuals often show prospecting: sampling several locations to compare habitat before making the choice. This is more likely to arise in species that possess the mobility and perceptive capabilities to perform such prospecting. This rather costly endeavour (Bonte et al., 2012; Jacob, Bestion, Legrand, Clobert, & Cote, 2015) is offset by the possible efficiency of this mechanism in increasing fitness (Berner & Thibert‐Plante, 2015). If the local fitness assessment is accurate, this mechanism of habitat choice will invariably increase fitness prospects and will by definition avoid an ecological trap. Moreover, matching habitat choice should be able to evolve to be more or less attracted to more suitable habitat.

Habitat choice is pervasive in plant–arthropod interactions. Indeed, an estimated 90% of phytophagous arthropods are specialized to a limited range of host plants, from feeding and ovipositing on multiple species in multiple families to highly specializing on one plant part of one plant species (Bernays & Graham, 1988; Jaenike, 1990; Schoonhoven, van Loon, & Dicke, 2005). Because of the typical intense relation of plant and phytophagous arthropod, possible runaway selection of plant defence and herbivore countermeasures promote specialization in arthropod herbivores. Furthermore, local host‐plant abundance, host plants as enemy‐free spaces, interspecific competition and plant‐associated assortative mating can drive host‐plant specialization (Price, Denno, Eubanks, Finke, & Kaplan, 2011). To find a suitable host plant in natural systems, which are often patchy and heterogeneously distributed, many phytophagous arthropods apply habitat choice in one form or another. (a) Direct genetic habitat choice mechanisms are very suitable for plant–arthropod systems. The large selective cost for ending up on the wrong host plant, applicable to specialized herbivores, is an important requirement for direct genetic habitat choice mechanisms to evolve. (b) Induced habitat choice is prevalent and effective because of the typical life cycle of arthropod herbivores. In many species, individuals develop on one plant and only disperse when turning adult. Therefore, habitat choice for an oviposition site is choosing the developmental habitat for its offspring (Gripenberg, Mayhew, Parnell, & Roslin, 2010). Since successful development to maturity is a prerequisite to reproducing and success on a particular host is heritable to some extent, a reproducing individual choosing to oviposit on the same host plant as where it successfully developed will oviposit on a suitable host for its offspring (Jermy, Hanson, & Dethier, 1968; Singer, Ng, & Thomas, 1988). (c) The case for matching habitat choice is less strong since many studies report a strong but not perfect correlation between host‐plant preference and performance (Gripenberg et al., 2010). Moreover, it is relatively hard to distinguish matching habitat choice from the other mechanisms. While an increase in fitness is the particular proximate driver of matching habitat choice, a fitness‐increasing pattern should not be exclusive to this mechanism because any choice should be adaptive at some point in order to evolve via natural selection. The strongest evidence for matching habitat choice comes from studies in which arthropod phenotypes were manipulated to decouple behavioural phenotype–environment matching from the genotype and previous experiences in terms of crypsis and thermoregulation (Edelaar et al., 2019; Gillis, 1982; Karpestam, Wennersten, & Forsman, 2012).

Here, we study the evolvability of habitat choice and the mechanisms involved in a *Tetranychus urticae* (two‐spotted spider mite) population. We report on an artificial selection experiment where we select populations making a habitat choice between tomato and cucumber plants. We selected each generation for ten generations according to three habitat choice selection treatments. If evolvable, we expect our particular selection regime to evolve habitat preferences, which results in an evolved choice, reflecting the selection treatment independent of the habitat choice mechanisms involved in *T. urticae.* Additionally, we test the contribution of different habitat choice mechanisms involved in host choice. While the initial choice for cucumber did not evolve over ten generations of artificial selection, quantifying life‐history traits before and after the experiment revealed that the mites adapted to tomato relative to cucumber. This now maladaptive habitat choice for cucumber is one of the first accounts of an ecological trap supported with information on preference and fitness before and after the onset of the trap and with insights on the choice mechanisms involved. Our experiment can add perspective to maladaptive choices in similar agricultural pest systems and broader ecology‐driven systems such as conservation, agriculture and evolutionary medicine.

## 2 MATERIALS AND METHODS

### 2.1 Experimental system

We performed the artificial selection experiment on a tomato‐bred *Tetranychus urticae* (two‐spotted spider mite) population. *T. urticae* is a cosmopolitan phytophagous mite species, a generalist best known as a pest in greenhouses. The mites have a sexual mating system with unfertilized females still able to produce males: arrhenotoky. Furthermore, it is also frequently used as model species in evolution and ecology (Alzate, Bisschop, Etienne, & Bonte, 2017; Belliure, Montserrat, & Magalhaes, 2010; Magalhães, Blanchet, Egas, & Olivieri, 2009; Van Petegem et al., 2018) due to its ease of maintaining a population, its rapid population growth (upwards of 10 offspring per individual per day), its short generation time (8–15 days) and the extensive knowledge on this species. In particular, *T. urticae* is known to adapt to host plants in a matter of 15 generation (Magalhães et al., 2009). We bred the particular *T. urticae* population used in this experiment, the stock, on tomato prior to the experiment. While this population has been living in the laboratory for multiple generations, studies show that it is not unprecedented for *T. urticae* to be bred in a laboratory for an extended period and retain a relatively high genotypic/phenotypic variation (Magalhães et al., 2007).

We opted to test the mites to choose *Solanum lycopersicum* L. moneymaker (tomato) over *Cucumis sativus* L. tanja (cucumber). We did not include the choice of *Phaseolus vulgaris L.* (bean), the optimal host plant for our *T. urticae* population. In past experiments, we never observed a higher performance on other host plants when they were adapting to them compared to bean. Since we wanted to study how choice evolves in concert with local adaptation affecting relative suitability in the choices involved, bean could never evolve to be the less adaptive choice. We used tomato plants of five weeks old and cucumber plants of three weeks old to ensure large enough leaves. Additionally, we used bean plants in further trait tests that were two weeks old.

### 2.2 Artificial selection experiment for habitat choice

We performed artificial selection on habitat choice. First, we sampled 100 individuals from a stock of tomato‐bred *Tetranychus urticae.* We maintained this new population on a tomato plant to keep any adaptation to tomato and to keep a consistent developmental environment for any possible induced habitat choice. Following, we submitted this population to ten rounds of artificial selection over ten generations. We did this according to one of three different artificial selection treatments that we replicate four times. We selected for (a) habitat choice for tomato, (b) habitat choice for cucumber and (c) random choice. Note that, although breeding all mites on tomato will influence any induced habitat choice, we still expect this plastic response to evolve to prefer the previously experienced habitat more or less, respectively, in the tomato choice and cucumber choice treatment.

**FIGURE 1:**
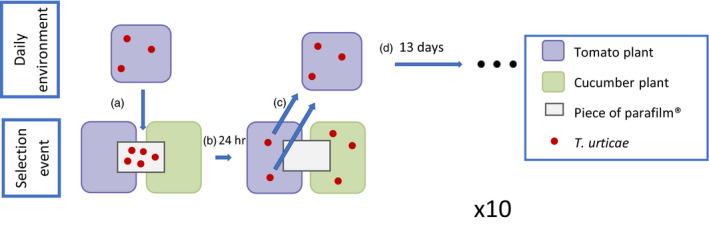
Schematic representation of an artificial selection round. For most of the time, mites live on a tomato plant. Mites are transferred to a choice arena (a), are given 24h to make a choice (b) and are selected according to the choice treatment, here tomato choice, and transferred to another tomato plant (c) where they breed the next generation (d). We repeated this for ten generations

One round of artificial selection included testing 100 females in a habitat choice arena for twenty‐four hours and breeding the selected next generation for the next selection round during the following thirteen days. From each replicate population, we transferred 100 random adult females on a piece of parafilm® (1 × 3 cm) on the intersection of a cucumber and tomato leaf both on life plants (Figure 1a). This piece of parafilm® provides a neutral, but inedible, surface from which the mites have to leave to be able to forage. When a mite moved to one of the two possible leaves, it could still move freely to the other without crossing the piece of parafilm® as to impair the mite's choice as little as possible. After twenty‐four hours, determined by pilot experiments and Egas and Sabelis (2001), we recorded the distribution of the mites between the two plants (Figure 1b), collected all mites that made the correct choice according to their selection treatment and placed them on a fresh tomato plant to breed the next generation (Figure 1c). This implies that we always collected mites from the tomato plant in the tomato choice treatment (1), always collected mites from the cucumber plant in the cucumber choice treatment (2) and collected mites from the tomato plant on odd rounds and from the cucumber plant on even rounds of the artificial selection in the random choice treatment (3). The alternation of the selected choice, selects against a consistent choice for one host plant. In preliminary trials, we observed that mites placed in a similar habitat choice arena wander a lot between the different choices the first few hours, likely prospecting, but do less so afterwards while they start feeding and laying eggs. Therefore, constraining the habitat choice period to twenty‐four hours ensured enough time for the mites to make a choice while constraining the time for the mites to eat the leaf considerably, which would drive a switch to the other host, and the time for local adaptation to differentially adapt the different selection regimes to both hosts. Thirteen days later, the new generation should reach adulthood, at which point we started a next round of artificial selection (Figure 1d). We maintained all replicates in high plastic boxes in a controlled environment (L:D 16‐hr/8‐hr photoperiod, 26–30°C).

### 2.3 Traits

#### 2.3.1 Life‐history traits

We quantified individual fertility and reproductive success on different host plants to estimate the level of adaptation to a host plant and the adaptive nature of a possible habitat choice. We tested this on tomato and cucumber, both plants involved in the artificial selection, but also on bean. We consider bean to be the optimal host of *T. urticae.* Comparing life‐history traits on tomato and cucumber to that on bean puts these in perspective of the expected optimal fitness.

We transferred a fertilized female from the population we aim to test to a bean leaf (15.75 cm^2^). On this common garden, the female produced the next generation that is controlled for effects of the maternal environment and effects of developmental plasticity. We placed females in their first day of adulthood on an empty patch of the tested host plant (1.5 × 2.5 cm, lined with paper towel strips). For each experimental population, we started three such common gardens, each from one female, and for every common garden, we tested one female offspring on bean, one female offspring on cucumber and one female offspring on tomato. We recorded the number of eggs laid within the first six days, henceforth fertility, and the number of female deutonymph offspring (i.e. life stage before adulthood) produced the first twelve days by the tested female, here referred to as reproductive success.

We tested these life‐history traits at the start of the experiment by introducing eight females from the tomato‐bred stock population to a common garden each. Furthermore, we tested this after the tenth round of the selection experiment by introducing three females from each of the twelve replicated populations to a common garden as explained above. Additionally, we tested the stock population at the end as a control by introducing eight females from the stock population to a common garden. This stock population was maintained under environmental conditions similar to the selection setups (L:D 16‐hr:8‐hr photoperiod, 27°C) on four touching tomato plants. Except for the occasional watering and replacement of roughly one old plant every week, this stock was not interfered with.

#### 2.3.2 Induced habitat choice

In order to test to what extent habitat choice is induced, we performed an additional induced habitat choice test. We distinguish induced habitat choice mechanisms from that of the other habitat choice mechanisms by varying the developmental environment of the tested populations. For that, we placed five fertilized females on a tomato leaf (15.75 cm^2^) and five fertilized females on a cucumber leaf (15.75 cm^2^) to develop a next generation. From each of these leaves, we placed twelve adults on a habitat choice arena. Such an arena is a petri dish (9 cm) with wet cotton on which a tomato and cucumber‐leaf patch is placed, touching each other (both 1.5 × 2.5cm). Paper towel strips line the two patches at their nontouching edges. The twelve mites were placed on a piece of parafilm® on the intersection of the two patches. After twenty‐four hours, we recorded the distribution of the twelve mites over the two patches and calculated the proportion of mites found on tomato. We performed this test on the stock population of tomato‐bred mites before the experiment, with which we started the artificial selection experiment, with six replicates for each developmental environment. We repeated this after the artificial selection experiment with one test for each of the twelve replicated populations on each developmental habitat.

An average proportion of 0.5 of mites found on tomato afterwards suggests no net habitat choice effect. A systematic deviation of the proportion of mites found on tomato from 0.5 in both developmental habitats results from noninduced habitat choice mechanisms, namely direct genetic habitat choice and matching habitat choice. Systematic differences in habitat choice between developmental habitats result from an induced mechanism.

### 2.4 Statistical models

We modelled our data using Bayesian inference. By using the “brms” (Bürkner, 2018) package in R that implements the statistical modelling platform “brms” (Carpenter et al., 2017). Posterior distributions of statistical model parameters were determined by Hamiltonian Monte Carlo (HMC) estimation. We modelled habitat choice data using a beta‐binomial distribution and counts for life‐history data using a gamma–Poisson distribution. Both account for overdispersion in the data and are the distributions with the biggest “entropy”, the most conservative distributions obeying the constraints of the data, for the respective data. Nonetheless, we tested each model with alternative distributions using the Watanabe–Akaike information criterion (WAIC) which supported the use of the aforementioned distributions. We also used WAICs to compare two models on the habitat choice during the selection rounds.

We modelled the log odds for habitat choice during the artificial selection rounds to depend linearly on time, that is the number of selection rounds, and on selection regime in both intercept and slope (in time) with an additional varying intercept and slope of each replica within a selection regime. We alternatively modelled this as only depending on the selection regime with a random intercept for each replica within it. We modelled the log of our reproductive success and fertility data to depend on the tested host plant and with a varying intercept for each replicated population in the life‐history trait tests after the selection rounds. We equally estimated the dependence of the log odds for habitat choice during the habitat imprinting tests on the developmental habitat. Details on the models, their structure and their priors can be found in the supplementary materials (see Appendix S1). We plot posterior predicted distributions with their 0.09, 0.50 and 0.91 percentile indicated to aid interpretation, not as a confidence threshold.

Our approach circumvents the assumptions and epistemological confusion that accompanies null‐hypotheses significance testing. This Bayesian approach allows for a more complete and nuanced appreciation of our results by (a) emphasizing estimated model parameters and resulting estimated dependent variables that are (b) expressed as likelihood distributions: the posterior distribution. Overall, we and a growing part of the research community deem Bayesian inference more appropriate because of its analogy with how ecological and evolutionary research is practiced (Pigliucci, 2002).

Data and scripts to the statistical models are available at https://github.com/fremorti/Trapped‐by‐habitat‐choice.

## 3 RESULTS

### 3.1 Artificial selection

**FIGURE 2:**
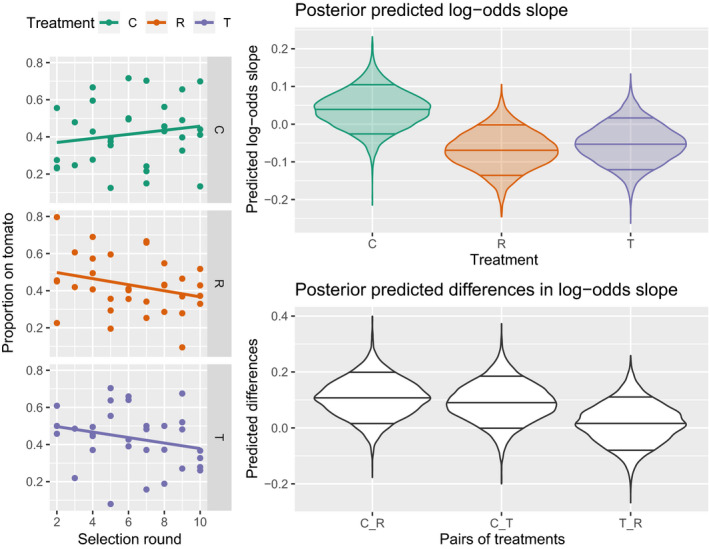
Tomato choice during the ten rounds of artificial selection. Left: proportion of mites ending up on tomato each selection round of populations selected for cucumber choice (C), random choice (R) and tomato choice (T). The lines plot a linear approximation of the fitted logistic model. Right: distribution of the posterior predicted log‐odds slopes (top) and pairwise differences in log‐odds slopes (bottom) of the HMC model on the data with the 0.09, 0.5 and 0.91 quantile indicated

We expected a higher tomato preference to evolve in the tomato choice treatment, a lower tomato preference in the cucumber choice treatment and no change in tomato preference in the no choice treatment. However, we recorded no convincing change in the proportion of mites ending up on the tomato plant during artificial selection when given the choice (Figure 2). Posterior predicted slopes displayed a wide spread, including zero (Figure 2, top right), and differences in posterior slopes showed no convincing differences between selection treatments (Figure 2, bottom right). Habitat choice did not change in a concise direction in any of the selection treatments. Furthermore, this is supported when we compare this model estimation with the same model but lacking the effect of time. A comparison of WAICs allocates a weight of 0.9 (WAIC = 775.4 ± 11.1 *SE*) to the model lacking the effect of time compared to 0.1 (WAIC = 777.8 ± 11.1 *SE*) to the initial model including the effect of time.

**FIGURE 3:**
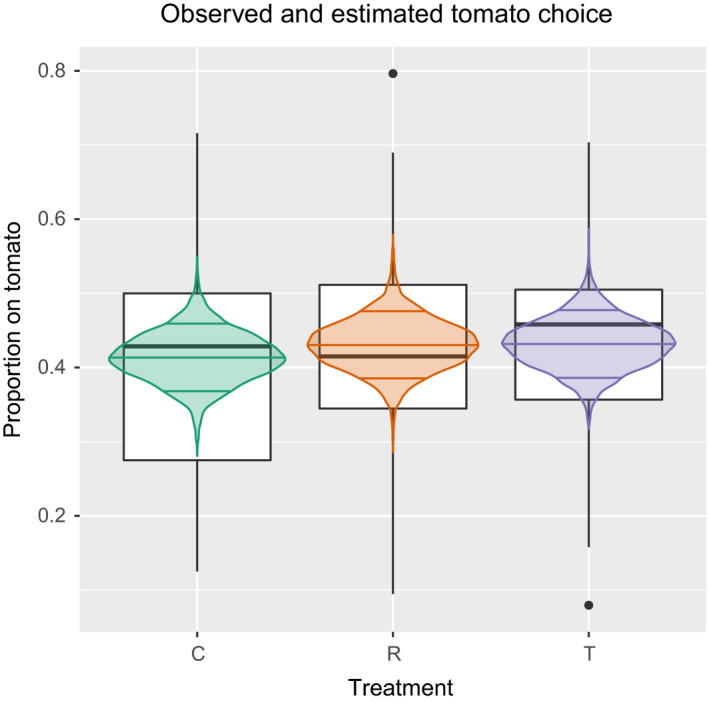
Tomato choice during the ten rounds of artificial selection with the proportion of mites ending up on tomato each selection round of populations selected for cucumber choice (C), random choice (R) and tomato choice (T). The boxplots plot the data and the violin plot plots the posterior predicted tomato choice for each treatment by the HMC model with the 0.09, 0.5 and 0.91 quantile indicated

While we do not record a change in the proportion of mites ending up on tomato during the selection rounds, we do record an overall lower likelihood to end up on tomato than on cucumber over all generations (Figure 3). Consequently, habitat choice did not evolve in response to the imposed selection but some form of habitat choice for cucumber was clear during the selection rounds.

#### 3.1.1 Life‐history traits

**FIGURE 4:**
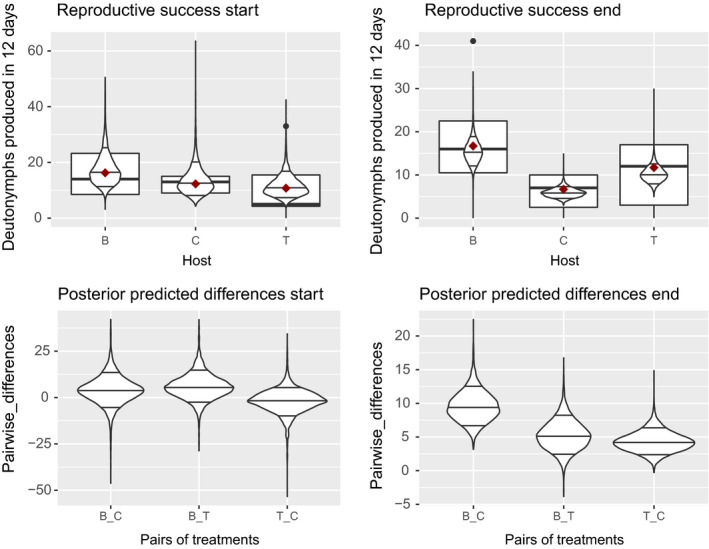
Top: reproductive success measured as number of deutonymphs produced by one female in twelve days on bean (B), cucumber (C) and tomato (T). The boxplot plots the data, the red dot the arithmetic mean of the data and the violin plot plots the posterior predicted reproductive success by the HMC model with the 0.09, 0.5 and 0.91 quantile indicated. Bottom: the posterior predicted pairwise differences in reproductive success. We tested this in the population used at the start of the experiment (left) and in all experimental and stock populations at the end of the experiment (right)

Reproductive success of the starting population was similar on tomato compared to cucumber at the start of the selection experiment (Figure 4, left). We observed the same pattern for fertility (see Appendix S2). At the end of the experiment, however, all replicated populations and our maintained stock population showed some degree of decreased local adaptation to cucumber relative to tomato as expressed by fertility (see Appendix S3 and S4) and reproductive success (Figure 4, right; Appendix S3). This suggests that a nonadaptive process, for example drift, decreased performance on cucumber but that the further exposure to tomato during the experimental duration has imposed further adaptive evolution to compensate or prevent a decrease of adaptation to tomato.

We detected no differences in the strength of adaptation in relation to the imposed selection treatments (see Appendix S3). However, a general decrease in adaptation compared to the stock population suggests that decreased performances (see Appendix S3) can be explained by evolutionary drift, likely caused by low effective population sizes and strong selection in the experimental populations.

#### 3.1.2 Induced habitat choice

**FIGURE 5:**
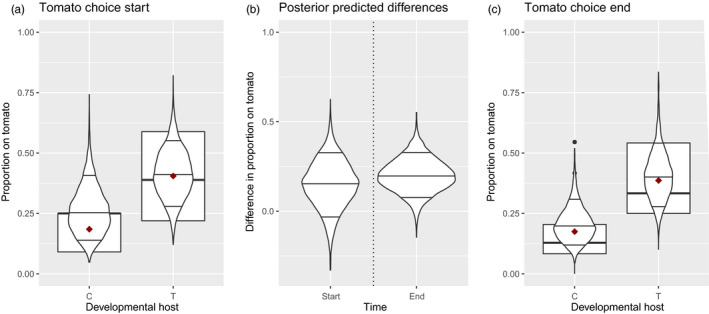
Tomato choice during induced habitat choice tests for mites developed on cucumber (C) and tomato (T) tested in the population used at the start of the experiment (a) and in all experimental populations at the end of the experiment (c). The boxplot plots the data, the red dot the arithmetic mean of the data and the violin plots the posterior predicted tomato choice by the HMC model with the 0.09, 0.5 and 0.91 quantile indicated. Posterior predicted differences in tomato choice between mites from both developmental hosts (b) at the start (left) and end (right) of the experiment

The likelihood interval of proportions of mites ending up on tomato at the end of the experiment was below 0.5 for mites reared on cucumber and predominantly below 0.5 in mites reared on tomato (Figure 5C). This indicates a tendency to choose cucumber despite tomato being the most suitable host at the end of the selection experiment. Furthermore, we detected a clear change in the mite's choice in response to the developmental environment. Mites developing on tomato tended to end up on tomato more frequently than mites developing on cucumber did (Figure 5B, right). The result from the habitat choice test before the start of the artificial selection experiment was similar but with a less convincing difference. Smaller sample sizes of this test resulted in larger uncertainty in the posterior predictions, a sizeable proportion of the posterior predictions of both developmental hosts higher than 50% (Figure 5A) and the difference in predicted posteriors now also including 0 (Figure 5B, left). In parallel with habitat choice during the selection rounds, this indicates that habitat choice and its induced portion did not evolve in response to the imposed selection treatments.

We detected no noteworthy differences in the overall and induced tomato choice between population of different selection treatments (see Appendix S4).

## 4 DISCUSSION

We experimentally demonstrated habitat choice in *Tetranychus urticae*. In the induced habitat choice test and the artificial selection rounds, we observed a tendency to choose cucumber over tomato (see Egas and Sabelis (2001) and Magowski, Egas, and Bruin (2003) but we were unable to evolve the underlying preferences. To explain the unexpected lack of evolution, we look at the requirements for evolution due to natural selection (Lewontin, 1970): trait variation, selection pressure resulting in a fitness difference between trait variants and for these fitness differences to be heritable. Our experiment was designed in a way to have the strongest selection pressure possible: all individuals making the right choice pass to the next generation, the others do not. While we do not have any information on how habitat choice is inherited from parent to offspring in *Tetranychus urticae*, the relative consistent choice for cucumber during the selection rounds and in the induced habitat choice tests would suggest at least some process of inheritance of traits involved. That would leave a lack in trait variation, especially habitat preference traits that would result in a difference in habitat choice. This could be the result of natural selection fixing a habitat preference trait in a past environment, or sustained lower population sizes and bottlenecks before or during the experiment eliminating variants in a stochastic way. As the same processes shape many natural systems, the experimental circumstances that may have hindered evolution do not prevent generalization of our results.

Notwithstanding, the induced habitat choice test hints at multiple habitat choice mechanisms. As mites choose cucumber more when they developed on it, an induced contribution to habitat choice is clear. Alternatively, this induced choice could be the result of phenotypic plasticity inducing a higher performance on the developmental environment combined with matching habitat choice, but as we will discuss, the mites do not appear to have a matching habitat choice. Additionally, mites did not have a net choice for tomato when developing on tomato, hinting at an additional noninduced contribution to a cucumber choice. While those tomato‐bred mites only show a slight, unconvincing trend to choose cucumber in the induced habitat choice test, mites during the experiment, who always developed on tomato as by the design of the experiment, showed a convincing choice for cucumber. Whether this noninduced contribution is due to direct genetic habitat choice or matching habitat choice is hard to say, but our data suggest the former. First, when considering the life‐history data, we notice that tomato is the equally suited host at the start or better suited host at the end. This means that the cucumber choice we observed is maladaptive. Negative matching habitat choice is very unlikely to evolve as this can never be an evolutionary stable strategy (Berner & Thibert‐Plante, 2015). Second, we observed a decrease in reproductive success on cucumber relative to the other hosts over the course of the ten artificial selection rounds. This decrease did not shift the preference for cucumber over time, which we expect under matching habitat choice. Both the maladaptive habitat choice and the unchanged preference with changing relative fitness prospects in both habitats are more likely under direct genetic habitat choice mechanisms. While we interpret tomato‐developed mites as slightly choosing cucumber, we admit that only a formal test with mites developed on a third host could have ruled out the alternative explanation of only an induced choice and only on cucumber. In short, we can confidently conclude that *Tetranychus urticae* has capabilities for habitat choice and that it involves induced habitat choice mechanisms. We also reasonably speculate that direct genetic habitat choice mechanisms contribute to this habitat choice.

Our experiments demonstrate that habitat choice is not always adaptive. The resulting choice from direct genetic habitat choice mechanisms and induced habitat choice mechanisms in our system lead the mites to poorer quality habitat in terms of fitness prospects, ecological traps (Hale & Swearer, 2016; Robertson & Hutto, 2006). While observations of maladapted populations are often attributed to an ecological trap without critical information on the behavioural preferences involved in moving, our experiment provides the essential mechanistic approach to habitat choice to conclude the presence of an ecological trap (Hale & Swearer, 2016). Moreover, our experiment provides an empirical account of the emergence of an ecological trap. A direct genetic habitat choice for cucumber in our mites most likely evolved by natural selection in a context where this choice was adaptive (see Egas & Sabelis, 2001; Gotoh, Bruin, Sabelis, & Menken, 1993), for example because of cucumber being more nutritious at that time or protecting mites more efficiently from predators in that environment. Our experimental environment and the subsequent adaptation to tomato changed the relative suitability of tomato and cucumber as hosts while the mite's preference to tomato remained unchanged. Corresponds with Brady's et al. (2019) moving target, which indicates maladaptation by the environment's optimum shifting away from the evolved preferences. However, the environment's optimum did not shift because of extrinsic changes in the hosts themselves but, rather, because of relative changes in adaptation to the hosts, with those changing traits as part of the environment from the point of view of the preference traits (genetic background).

In making ecological traps possible, induced and direct genetic choice mechanisms constrain how likely habitat choice can rescue populations endangered by environmental change. Habitat choice, when adaptive, should enable a population to survive in a changing environment by cueing in on optimal habitat. If contemporary change is rapid, habitat choice potentially causes an ecological trap by hampering populations to escape now unsuitable habitat and colonize more suitable habitat. This puts endangered populations even more at the brink of extinction rather than rescuing them. First of all, matching habitat choice mechanisms are not expected to cause an ecological trap due to the direct connection of preference and performance unless performance assessment is inaccurate for some reason. Second, a trap emerged from direct genetic choice mechanisms can be very stable since those mechanisms are more conservative and less likely to evolve. Third, a trap emerged from induced habitat choice mechanisms is less stable since maladaptive choice for the still preferred habitat, assuming some imperfection, will still result in some individuals ending up on the more adaptive habitat that is not preferred. As a result, those individual's offspring will predominantly choose the adaptive habitat as a result of their induced preference for it. Because of differences in reproductive success between both habitats, the proportion of individuals across the landscape that experience induced choice mechanisms as adaptive will grow over time and the ecological trap will fade. Still, the ecological damage done during the time of the ecological trap could prove detrimental. Evolution of habitat choice that is rapid enough to minimize the lag behind the changing performance is anticipated to rescue populations from such an ecological trap. Such a rapid adaptation can, however, only be achieved when heritable variation in preference is substantial. This insight provides opportunities to manage the likelihood of ecological traps emerging in (semi‐)natural systems. Our experiment resembles an agricultural system where our focal species *T. urticae* or an ecologically similar pest species causes massive damage. Managing the evolvability of habitat preferences could spring an ecological trap to control such systems more easily. Someone interested in doing so could manage the available variation in habitat preferences or manage the selection pressure on habitat preferences. For instance, one could think of shifting plant preference of the pest in your agricultural system by introducing a better host. A subsequent extermination, if unsuccessful, would still constrain evolvability in habitat preference where the introduced host can function as an ecological trap. More generally, conservation efforts may need to preserve the evolvability of habitat preferences in an endangered population by putting an even higher priority on increasing population sizes (Derry et al., 2019). Otherwise, conservators need to preserve the suitability of the preferred habitat to prevent an ecological trap or other forms of maladaptation.

However, there even are evolutionary constraints of adaptive habitat choice to cope with a changing environment. Habitat choice, as evolved behaviour, is expected to promote local adaptation and specialization (Armsworth, 2009; Holt, 1987; Jacob et al., 2017; Nicolaus & Edelaar, 2018; Ravigné et al., 2009), thereby promoting niche conservatism (Futuyma & Moreno, 1988; Holt, 1987; Holt & Barfield, 2008) that reduces the opportunity for natural selection to evolve adaptation to less‐optimal habitat. When environmental change in the landscape is persistent, the temporary compensation of fitness decline by choosing optimal habitat could prove detrimental in the end when this eliminates the opportunity for adaptation to the deteriorating habitat. However, some theoretical findings suggest that matching habitat choice can stimulate rather than constrain adaptation to deteriorated habitat when adaptation to suboptimal habitat is facilitated by specific single mutations (Holt & Barfield, 2008).

In summary, we show the presence of habitat choice in a laboratory population of *Tetranychus urticae* (two‐spotted spider mite). This habitat choice involves induced habitat choice mechanisms and likely involves a direct genetic habitat choice mechanism. Our experiment reveals the emergence of an ecological trap. This important but predominantly theoretically approached process can cause habitat choice to be a maladaptation rather than an adaptation and constrain its ability to rescue populations from environmental challenges.

## CONFLICT OF INTEREST

None declared.

## Supporting information

Supplementary Material

## Data Availability

Data and scripts to the statistical models are available at https://github.com/fremorti/Trapped‐by‐habitat‐choice, reference number 179,680,661.
