# Supplementary material for: Trapped by habitat choice: Ecological trap emerging from adaptation in an evolutionary experiment
Source: Evol Appl. 2020 Mar 28;13(8):1877–87. doi: 10.1111/eva.12937 (PMC7463321; doi:10.1111/eva.12937)
Supplement: Supplementary file 1 — Supplementary Material [file EVA-13-1877-s001.zip › eva12937-sup-0001-AppendixA.docx]

## Appendix A: Statistical model description and estimates

All models were run in R using brms and Hamiltonian Monte Carlo with two chains with each 5000 iteration from which 2000 were warmup

Model 1.1: Tomato preference during the ten rounds of artificial selection.

1. Model representation

$$P \sim Betabinomial\left( n, prop, \theta\right)$$

$$logit\left( prop \right)= a_{rep\left[ i \right]}+aC*treatC+aT*treatT+round*(b_{rep\left[ i \right]}+bC*treatC+bT*treatT)$$

$$\left[ \begin{matrix} a_{rep\left[ i \right]} \\ b_{rep\left[ i \right]} \end{matrix} \right] \sim MVNormal\left( \left[ \begin{matrix} a_{0} \\ b_{0} \end{matrix} \right], S \right)$$

$$S= \left( \begin{matrix} \sigma_{a} & 0 \\ 0 & \sigma_{b} \end{matrix} \right) R \left( \begin{matrix} \sigma_{a} & 0 \\ 0 & \sigma_{b} \end{matrix} \right)$$

$$a_{0} \sim Normal\left( 0, 2 \right)$$

$$b_{0} \sim Normal\left( 0, 2 \right)$$

$$aC \sim Normal\left( 0, 2 \right)$$

$$aT \sim Normal\left( 0, 2 \right)$$

$$bC \sim Normal\left( 0, 2 \right)$$

$$bT \sim Normal\left( 0, 2 \right)$$

$$\sigma_{a} \sim Cauchy\left( 0, 1 \right)$$

$$\sigma_{b} \sim Cauchy\left( 0, 1 \right)$$

$$R \sim LKJCorr\left( 2 \right)$$

$$\theta\sim Exp\left( 1 \right)$$

With *P* the proportion of mites preferring tomato, *n* the sample size, *rep[i]* the i^th^ replicate, *treatC* indicating the cucumber choice selection treatment, *treatT* indicating the tomato choice selection treatment and *round* the round or generation number. Betabinomial, MVNormal, Normal, Cauchy, LKJCorr, Exp and GammaPoisson, respectively, indicate a beta-bionomial, multivariate normal, normal, cauchy, Lewandowski Kurowicka Joe correlation matrix, exponential and gamma-Poisson distributions in this and following models. The LKJ correlation distribution is a prior for the correlation matrix for varying intercepts and varying slopes.

We choose relatively strong regularizing priors that make sense when modelling habitat preferences. The prior for intercepts, slopes and their deviations represents log-odds for preference that are mostly between -4 and 4 (two standard deviations, or almost 96% of that distribution) and roughly transform to a preference between 2% and 98%. We do not expect extremer preferences or changes in preference from the fact that the mites have no (known) evolutionary history on these hosts and from previous experience with mites on these hosts.

1. Model parameter estimates

mean se_mean sd 2.5% 97.5% n_eff Rhat

b_Intercept -0.58 0.01 0.32 -1.23 0.05 3482 1

b_treatmentR 0.72 0.01 0.45 -0.17 1.63 3880 1

b_treatmentT 0.62 0.01 0.46 -0.28 1.51 4076 1

b_selection 0.04 0.00 0.05 -0.06 0.14 3419 1

b_treatmentR:selection -0.11 0.00 0.07 -0.24 0.02 3816 1

b_treatmentT:selection -0.09 0.00 0.07 -0.23 0.05 3899 1

sd_code__Intercept 0.14 0.00 0.11 0.01 0.42 3558 1

sd_code__selection 0.02 0.00 0.02 0.00 0.06 3962 1

cor_code_Interc_selection -0.13 0.01 0.46 -0.88 0.76 8061 1

phi 8.51 0.01 1.33 6.17 11.37 10273 1

r_code[1,Intercept] -0.04 0.00 0.15 -0.41 0.25 6877 1

r_code[2,Intercept] -0.06 0.00 0.16 -0.46 0.20 6028 1

r_code[3,Intercept] 0.03 0.00 0.15 -0.26 0.39 5673 1

r_code[4,Intercept] 0.01 0.00 0.15 -0.30 0.33 6830 1

r_code[5,Intercept] 0.02 0.00 0.15 -0.27 0.37 6477 1

r_code[6,Intercept] -0.04 0.00 0.15 -0.41 0.23 5547 1

r_code[7,Intercept] -0.02 0.00 0.15 -0.36 0.29 5917 1

r_code[8,Intercept] 0.05 0.00 0.15 -0.21 0.45 5784 1

r_code[9,Intercept] -0.05 0.00 0.15 -0.42 0.21 6052 1

r_code[10,Intercept] 0.02 0.00 0.15 -0.28 0.37 6235 1

r_code[11,Intercept] 0.04 0.00 0.15 -0.25 0.39 6116 1

r_code[12,Intercept] 0.03 0.00 0.15 -0.26 0.42 5871 1

r_code[1,selection] 0.00 0.00 0.02 -0.05 0.04 6080 1

r_code[2,selection] 0.00 0.00 0.02 -0.05 0.04 5943 1

r_code[3,selection] 0.00 0.00 0.02 -0.05 0.04 5551 1

r_code[4,selection] 0.00 0.00 0.02 -0.04 0.05 6170 1

r_code[5,selection] 0.00 0.00 0.02 -0.05 0.04 5517 1

r_code[6,selection] 0.00 0.00 0.02 -0.05 0.04 5347 1

r_code[7,selection] 0.00 0.00 0.02 -0.05 0.04 5637 1

r_code[8,selection] 0.00 0.00 0.02 -0.04 0.05 5572 1

r_code[9,selection] 0.00 0.00 0.02 -0.05 0.03 5108 1

r_code[10,selection] 0.00 0.00 0.02 -0.03 0.05 6200 1

r_code[11,selection] 0.01 0.00 0.02 -0.03 0.06 5349 1

r_code[12,selection] 0.00 0.00 0.02 -0.03 0.06 5892 1

Model 1.2: Alternative model for tomato preference during the ten rounds of artificial selection without the temporal effect.

1. Model representation

$$P \sim Betabinomial\left( n, prop, \theta\right)$$

$$logit\left( prop \right)= a_{rep\left[ i \right]}+aC*treatC+aT*treatT$$

$$a_{rep\left[ i \right]} \sim Normal\left( a_{0},\sigma_{a} \right)$$

$$a_{0} \sim Normal\left( 0, 2 \right)$$

$$aC \sim Normal\left( 0, 2 \right)$$

$$aT \sim Normal\left( 0, 2 \right)$$

$$\sigma_{a} \sim HalfCauchy\left( 0, 1 \right)$$

$$\theta\sim Exp\left( 1 \right)$$

1. Model parameter estimates

mean se_mean sd 2.5% 97.5% n_eff Rhat

b_Intercept -0.35 0.00 0.14 -0.62 -0.07 3929 1

b_treatmentR 0.07 0.00 0.20 -0.32 0.45 4476 1

b_treatmentT 0.07 0.00 0.20 -0.31 0.47 4006 1

sd_code__Intercept 0.11 0.00 0.09 0.00 0.32 2544 1

phi 8.32 0.02 1.25 6.10 10.98 6710 1

r_code[1,Intercept] -0.04 0.00 0.12 -0.33 0.17 3517 1

r_code[2,Intercept] -0.05 0.00 0.13 -0.38 0.16 4407 1

r_code[3,Intercept] 0.01 0.00 0.11 -0.22 0.28 5935 1

r_code[4,Intercept] 0.01 0.00 0.11 -0.23 0.26 5998 1

r_code[5,Intercept] 0.01 0.00 0.11 -0.22 0.27 5216 1

r_code[6,Intercept] -0.03 0.00 0.12 -0.34 0.20 5495 1

r_code[7,Intercept] -0.02 0.00 0.12 -0.29 0.22 4374 1

r_code[8,Intercept] 0.05 0.00 0.12 -0.16 0.35 3971 1

r_code[9,Intercept] -0.04 0.00 0.12 -0.34 0.16 5655 1

r_code[10,Intercept] 0.03 0.00 0.11 -0.19 0.30 5081 1

r_code[11,Intercept] 0.04 0.00 0.12 -0.17 0.33 4201 1

r_code[12,Intercept] 0.04 0.00 0.12 -0.18 0.32 4336 1

Model 2.1: reproductive success test before experiment

1. Model representation

$$R \sim Gammapoisson\left( \lambda, \theta\right)$$

$$log\left( \lambda\right)= a+bC*treatC+bT*treatT$$

$$a \sim Normal\left( 0, 4 \right)$$

$$bC \sim Normal\left( 0, 4 \right)$$

$$bT \sim Normal\left( 0, 4 \right)$$

$$\theta\sim Exp\left( 1 \right)$$

With *R* the amount of deutonymphs produced by a female in twelve days, *treatC* indicates whether reproductive success was tested on cucumber, *treatT* indicates reproductive success tested on tomato (third host tested was bean). All priors are not very regularizing when considering them estimating log values of fertility.

We choose relatively uninformative priors when modelling the life-history traits that for the intercept and the deviations are mostly situated between -8 and 8, which transforms to 0.003 and 2980.

1. Model estimates

mean se_mean sd 2.5% 97.5% n_eff Rhat

b_Intercept 2.82 0.00 0.30 2.27 3.44 4167 1

b_patchC -0.27 0.01 0.45 -1.16 0.63 4330 1

b_patchT -0.41 0.01 0.43 -1.27 0.46 4219 1

shape 1.64 0.01 0.56 0.79 2.95 4148 1

Model 2.2: reproductive success test after experiment

1. Model representation

$$R \sim Gammapoisson\left( \lambda, \theta\right)$$

$$log\left( \lambda\right)= a_{trep\left[ i \right]}+bC*treatC+bT*treatT$$

$$a_{trep\left[ i \right]}\sim Normal(a, \sigma_{trep})$$

$$a \sim Normal\left( 0, 10 \right)$$

$$bC \sim Normal\left( 0, 5 \right)$$

$$bT \sim Normal\left( 0, 5 \right)$$

$$\sigma_{trep} \sim Cauchy\left( 0, 2 \right)$$

$$\theta\sim Exp\left( 1 \right)$$

With *R* the amount of deutonymphs produced by a female in twelve days, *rep[i]* the i^th^ replicated treatment, *treatC* indicates whether reproductive success was tested on cucumber and *treatT* indicates whether reproductive success was tested on tomato (third host tested was bean).

1. Model estimates

mean se_mean sd 2.5% 97.5% n_eff Rhat

b_Intercept 2.73 0.00 0.17 2.38 3.06 2878 1

b_patchC -0.97 0.00 0.17 -1.30 -0.65 6005 1

b_patchT -0.42 0.00 0.16 -0.75 -0.10 6299 1

sd_trep__Intercept 0.43 0.00 0.15 0.19 0.79 1666 1

shape 2.51 0.01 0.49 1.69 3.59 4114 1

r_trep[C1,Intercept] 0.09 0.00 0.24 -0.37 0.61 5216 1

r_trep[C2,Intercept] 0.04 0.00 0.24 -0.40 0.52 4913 1

r_trep[C3,Intercept] 0.39 0.00 0.27 -0.08 0.98 4016 1

r_trep[C4,Intercept] 0.11 0.00 0.24 -0.34 0.59 4906 1

r_trep[R1,Intercept] 0.21 0.00 0.26 -0.27 0.76 4864 1

r_trep[R2,Intercept] -0.67 0.01 0.38 -1.50 0.00 3359 1

r_trep[R3,Intercept] -0.39 0.00 0.27 -0.94 0.10 4055 1

r_trep[R4,Intercept] 0.11 0.00 0.23 -0.33 0.62 4416 1

r_trep[S13,Intercept] 0.49 0.00 0.20 0.11 0.90 3131 1

r_trep[T1,Intercept] 0.16 0.00 0.24 -0.31 0.66 4045 1

r_trep[T2,Intercept] 0.06 0.00 0.23 -0.38 0.55 4747 1

r_trep[T3,Intercept] -0.25 0.00 0.25 -0.74 0.23 5154 1

r_trep[T4,Intercept] -0.29 0.00 0.25 -0.79 0.19 5069 1

Model 3.1: habitat imprinting test before experiment

1. Model representation

$$P \sim Betabinomial\left( total, prop, \theta\right)$$

$$logit\left( prop \right)= a+b1*devT$$

$$a \sim Normal\left( 0, 2 \right)$$

$$bT \sim Normal\left( 0, 2 \right)$$

$$\theta\sim Exp\left( 1 \right)$$

With *P* the proportion of mites preferring tomato, *total* the sample size and *devT* indicating whether the preference was tested on mites developed on tomato (as opposed to cucumber). The same regularizing prior for the intercept and deviation on it is chosen as in the intercept in model 1 for the same reasons.

1. Model estimates

mean se_mean sd 2.5% 97.5% n_eff Rhat

a -1.08 0.01 0.53 -2.15 -0.06 4252 1

bT 0.73 0.01 0.66 -0.55 2.01 4450 1

θ 3.71 0.02 1.55 1.44 7.39 4425 1

Model 3.2: habitat imprinting test after experiment

1. Model representation

$$P \sim Betabinomial\left( total, prop, \theta\right)$$

$$logit\left( prop \right)= a+ b_{treatment[i]}+bC*devC$$

$$a \sim Normal\left( 0, 2 \right)$$

$$b_{treatment[i]} \sim Normal\left( b, \sigma\right)$$

$$b \sim Normal\left( 0, 2 \right)$$

$$\sigma\sim Normal\left( 0, 2 \right)$$

$$bT \sim Normal\left( 0, 2 \right)$$

$$\theta\sim Exp\left( 1 \right)$$

With *P* the proportion of mites preferring tomato, *total* the sample size, *devT* indicating whether the preference was tested on mites developed on cucumber (as opposed to tomato) and $b_{treatment[i]}$ the variable intercept of treatment i.

1. Model estimates

mean se_mean sd 2.5% 97.5% n_eff Rhat

a -1.40 0.01 0.43 -2.26 -0.53 1775 1

bT 1.00 0.01 0.45 0.13 1.91 3704 1

sd_treatment__Intercept 0.46 0.01 0.40 0.01 1.51 1424 1

θ 4.59 0.03 1.52 2.23 8.12 3691 1

b -0.92 0.01 0.36 -1.63 -0.16 1433 1

b[C,Intercept] -0.01 0.01 0.36 -0.81 0.76 2041 1

b[R,Intercept] 0.16 0.01 0.39 -0.55 1.09 1789 1

b[T,Intercept] -0.21 0.01 0.40 -1.23 0.48 1693 1
