# Supplementary material for: Trapped by habitat choice: Ecological trap emerging from adaptation in an evolutionary experiment
Source: Evol Appl. 2020 Mar 28;13(8):1877–87. doi: 10.1111/eva.12937 (PMC7463321; doi:10.1111/eva.12937)
Supplement: Supplementary file 1 — Supplementary Material [file EVA-13-1877-s001.zip › eva12937-sup-0002-AppendixB.docx]

## Appendix B: fertility


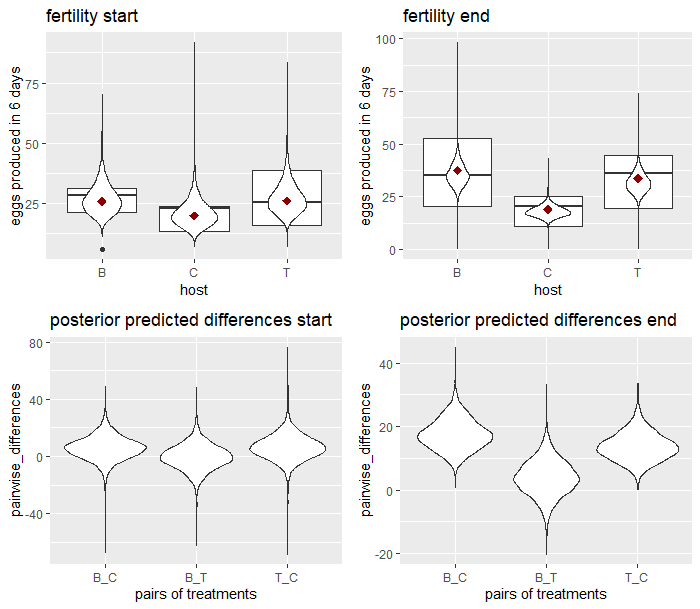


Figure B1. Top: fertility estimated as eggs produced by one female in six days on bean (B), cucumber (C) and tomato (T). The boxplot plots the data, the red dot the arithmetic mean of the data and the violin plots the posterior predicted fertility by the HMC model. Bottom: the posterior predicted pairwise differences in fertility. This was tested in the population used at the start of the experiment (left) and in all experimental and stock populations at the end of the experiment (right).

In parallel with reproductive success, fertility at the start did not suggest adaptation to tomato. The distributions of posterior predicted fertility on cucumber and tomato overlap notably (fig. B1, top left) and their posterior predicted differences in fertility overlap 0 very convincingly (fig. B1, bottom left). Additionally, fertility at the end also demonstrated mites to be tomato adapted by laying on average more eggs on tomato compared to cucumber (fig. B1, top right) as their differential response is convincingly different from 0 (fig. B1, bottom right).
