# Supplementary material for: Trapped by habitat choice: Ecological trap emerging from adaptation in an evolutionary experiment
Source: Evol Appl. 2020 Mar 28;13(8):1877–87. doi: 10.1111/eva.12937 (PMC7463321; doi:10.1111/eva.12937)
Supplement: Supplementary file 1 — Supplementary Material [file EVA-13-1877-s001.zip › eva12937-sup-0003-AppendixFigB1.pdf]

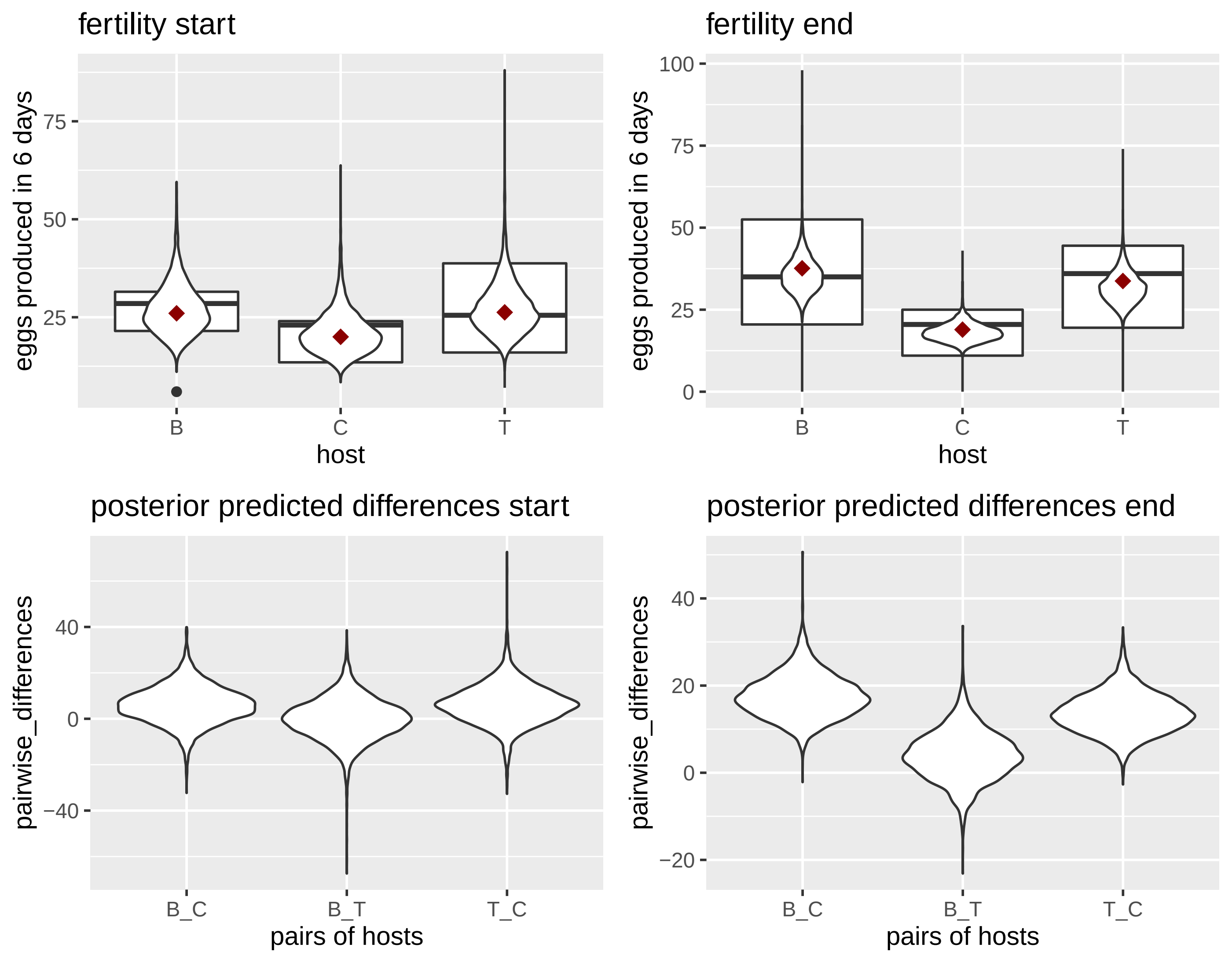

fertility start
eggs produced in 6 days
75
50
25
B
C
T
host
fertility end
eggs produced in 6 days
100
75
50
25
0
B
C
T
host
posterior predicted differences start
pairwise_differences
40
0
−40
B_C
B_T
T_C
pairs of hosts
posterior predicted differences end
pairwise_differences
40
20
0
−20
B_C
B_T
T_C
pairs of hosts
