# Supplementary material for: Trapped by habitat choice: Ecological trap emerging from adaptation in an evolutionary experiment
Source: Evol Appl. 2020 Mar 28;13(8):1877–87. doi: 10.1111/eva.12937 (PMC7463321; doi:10.1111/eva.12937)
Supplement: Supplementary file 1 — Supplementary Material [file EVA-13-1877-s001.zip › eva12937-sup-0004-AppendixC.docx]

## Appendix C: effect selection regime on life-history traits

Fertility or reproductive success on a host plant did not differ between experimental populations. This suggests that the different habitat choice selection regimes did not select for different levels of local adaptation on any of the host plants. However, we observed that fertility and reproductive success on all host plants is higher for the stock population compared to any of the experimental treatment (fig. C1, C2). This points at the importance of drift in the evolution of adaptation to the different host plants within our experimental setup due to relatively low population sizes and frequent bottlenecking.

Figure C1. Fertility estimated as eggs produced in six days on bean (left), cucumber (middle) and tomato (right) of the different experimental treatments: tomato choice (T), random choice (R), cucumber choice (C) and control (S) at the end of the experiment. The red dot indicates the arithmetic mean of the data.


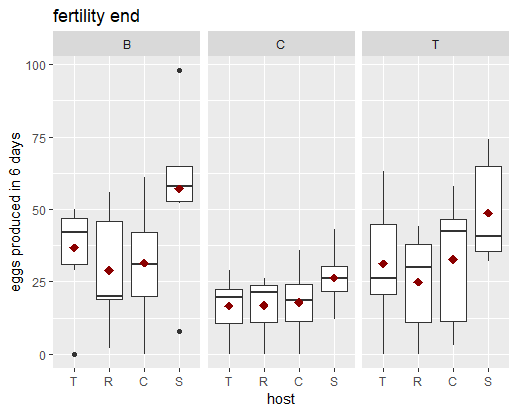

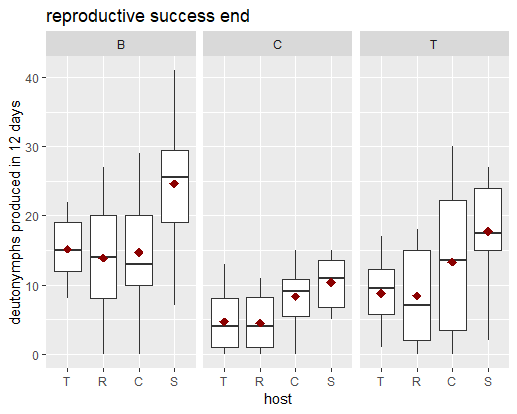


Figure C2. Reproductive success estimated as deutonymphs produced in twelve days on bean (left), cucumber (middle) and tomato (right) of the different experimental treatments: tomato choice (T), random choice (R), cucumber choice (C) and control (S) at the end of the experiment. The red dot indicates the arithmetic mean of the data.
