# Supplementary material for: Trapped by habitat choice: Ecological trap emerging from adaptation in an evolutionary experiment
Source: Evol Appl. 2020 Mar 28;13(8):1877–87. doi: 10.1111/eva.12937 (PMC7463321; doi:10.1111/eva.12937)
Supplement: Supplementary file 1 — Supplementary Material [file EVA-13-1877-s001.zip › eva12937-sup-0007-AppendixD.docx]

## Appendix D: effect selection regime on induced habitat choice


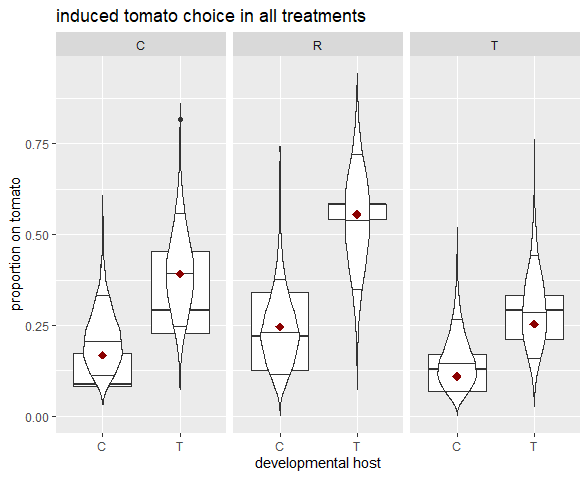


Figure D1. Tomato choice during induced habitat choice tests for mites originating from the cucumber choice treatment (left), random choice treatment (middle) and tomato choice treatment (right). Mites developed on cucumber (C) and tomato (T). The boxplot plots the data, the diamond the arithmetic mean of the data and the violin plots the posterior predicted tomato choice by the HMC model with the 0.09, 0.5 and 0.91 quantile indicated.

We tested the effect of the experimental treatment in concert with the developmental habitat and their interaction on tomato choice in the induced habitat choice test. We anticipated that the experimental treatment would affect the induced habitat choice because mites in our experimental populations were always induced for tomato habitat. Therefore, tomato choice populations would be selected for an increased induced effect and cucumber choice populations would be selected for a decreased induced effect. However, we observed no noteworthy differences in habitat choice induction between mites from different experimental populations (fig. D1). Choice slightly more induced in the random choice populations and slightly less in the tomato choice populations but the relatively small differences and large spread of the likelihood distributions does not indicated a meaningful treatment effect. Moreover, when comparing this model (WAIC = 102.2 ± 4.9 SE) to the one with only the developmental habitat as explaining variable (WAIC = 99.3 ± 4.9 SE) the latter is preferred completely (weight of 1) over the model discussed here.
