# Supplementary figures and images for: Trapped by habitat choice: Ecological trap emerging from adaptation in an evolutionary experiment
Source: Evol Appl. 2020 Mar 28;13(8):1877–87. doi: 10.1111/eva.12937 (PMC7463321; doi:10.1111/eva.12937)

# induced tomato choice in all treatments

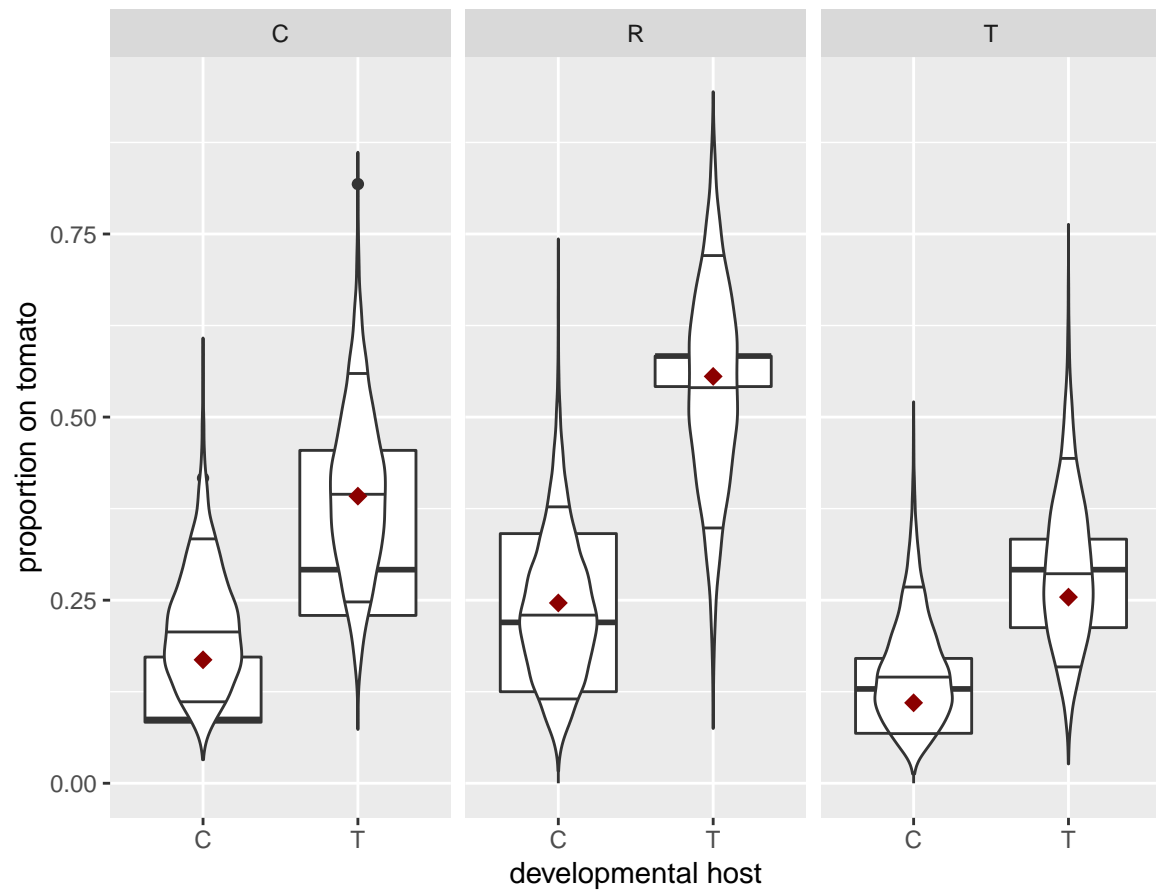

Supplement: Supplementary file 1 — Supplementary Material [file EVA-13-1877-s001.zip › eva12937-sup-0008-AppendixFigD1.pdf]
